# A Template Model Explains Jerboa Gait Transitions Across a Broad Range of Speeds

**DOI:** 10.3389/fbioe.2022.804826

**Published:** 2022-04-27

**Authors:** Jiayu Ding, Talia Y. Moore, Zhenyu Gan

**Affiliations:** ^1^ Dynamic Locomotion and Robotics Laboratory, Department of Mechanical and Aerospace Engineering, Syracuse University, Syracuse, NY, United States; ^2^ Evolution and Motion of Biology and Robotics Laboratory, Department of Mechanical Engineering, Robotics Institute, Ecology and Evolutionary Biology, and Museum of Zoology, University of MI, Ann Arbor, MI, United States

**Keywords:** legged robots, dynamics, bipedal locomotion, non-cursorial locomotion, gait transitions

## Abstract

For cursorial animals that maintain high speeds for extended durations of locomotion, transitions between footfall patterns (gaits) predictably occur at distinct speed ranges. How do transitions among gaits occur for non-cursorial animals? Jerboas (*Jaculus*) are bipedal hopping rodents that frequently transition between gaits throughout their entire speed range. It has been hypothesized that these non-cursorial bipedal gait transitions are likely to enhance their maneuverability and predator evasion ability. However, it is difficult to use the underlying dynamics of these locomotion patterns to predict gait transitions due to the large number of degrees of freedom expressed by the animals. To this end, we used empirical jerboa kinematics and dynamics to develop a unified spring Loaded Inverted Pendulum model with defined passive swing leg motions. To find periodic solutions of this model, we formulated the gait search as a boundary value problem and described an asymmetrical running gait exhibited by the jerboas that emerged from the numerical search. To understand how jerboas change from one gait to another, we employed an optimization approach and used the proposed model to reproduce observed patterns of jerboa gait transitions. We then ran a detailed numerical study of the structure of gait patterns using a continuation approach in which transitions are represented by bifurcations. We found two primary mechanisms to increase the range of speeds at which gait transitions can occur. Coupled changes in the neutral leg swing angle alter leg dynamics. This mechanism generates changes in gait features (e.g., touchdown leg angle and timings of gait events) that have previously been shown to induce gait transitions. This mechanism slightly alters the speeds at which existing gait transitions occur. The model can also uncouple the left and right neutral leg swing angle, which generates asymmetries between left and right leg dynamics. New gait transitions emerge from uncoupled models across a broad range of speeds. In both the experimental observations and in the model, the majority of the gait transitions involve the skipping and asymmetrical running gaits generated by the uncoupled neutral leg swing angle mechanism. This simulated jerboa model is capable of systematically reproducing all biologically relevant gait transitions at a broad range of speeds.

## 1 Introduction

Despite vast differences in morphology, the locomotion patterns of many legged animals are strikingly similar (Alexander, 2002). Typically, these gait patterns can be characterized by repeated footfall sequences (Alexander, 1984; Hildebrand, 1989), the ground reaction force profile (Alexander, 2009) or by how gravitational, potential and kinetic energies are exchanged over the course of a stride (Cavagna et al., 1977). As the speed of locomotion increases, quadrupedal cursorial animals, such as horses or gazelles, switch from using a walking gait at low speeds to a trotting or pacing gait at intermediate speeds, and then a galloping gait at their highest speeds. Previous studies suggest that each gait minimizes oxygen consumption (Hoyt and Taylor, 1981; Minetti et al., 1999) and minimizes the loading impact on the musculoskeletal system (Farley and Taylor, 1991; Lee et al., 2011) at a distinct speed range. Therefore, transitioning between gaits as speed increases helps cursorial animals minimize the cost of sustained steady-state locomotion, thereby enhancing endurance at high speeds. Based on these fundamental principles, the speeds at which cursorial gaits occur can be predicted by the ratio of centripetal to gravitational force (as an animal moves over its supporting limb), or the Froude number (Alexander and Jayes, 2009).

On the other hand, rapid and energetically costly changes in acceleration and direction of movement are important for small animals evading predators (Biewener and Blickhan, 1988; Chance and Russell, 2009; Domenici et al., 2011). Some quadrupedal and hexapedal prey animals temporarily rear up on hindlimbs and use bipedal locomotion to enhance acceleration during escape (Full and Tu, 1991; Clemente, 2014). Notably, jerboas (Dipodidae) are desert rodents that evolved obligately bipedal locomotion from quadrupedal ancestors. Although pentapedal (quadrupedal with additional support from the tail) locomotion occurs during in postnatal development (Eilam and Shefer, 1997), and quadrupedal locomotion is used infrequently at slower speeds (Happold, 1967), jerboas are the only hopping rodent to use multiple bipedal gaits as their primary mode of locomotion as adults (Moore et al., 2017). The hopping, skipping, and running gaits are used throughout the entire jerboa speed ranges, with frequent ( ≈50% of all recorded trials) transitions between gaits that are not predicted by the Froude equation (Moore et al., 2017). Because each gait is associated with a distinct range of acceleration, rather than speed, frequent gait transitions likely enhance the potential maneuverability and predator evasion ability of a jerboa (Moore et al., 2017). Thus, building models to characterize non-cursorial locomotion can help us understand more agile and maneuverable locomotion.

The center of mass dynamics and kinematics for a wide variety of cursorial animals can be modeled using a simplified “template” approach with minimal degrees of freedom (Full and Koditschek, 1999). McGeer (1990) demonstrated that an Inverted Pendulum model (IP) with two rigid legs is capable of walking on a sloped ramp without the help of any additional controllers or actuators. A Spring-Loaded Inverted Pendulum (SLIP) model explains the kinetic and potential energy exchanges in running gaits (Blickhan, 1989; Farley et al., 1993). These models have been shown to explain the locomotion of cursorial animals that differ greatly in size, leg number, or posture. The simplicity and broad applicability of these template models have made them invaluable for designing controllers for legged robots (Hereid et al., 2014; DSCC, 2015).

Although these simplified models have been useful for generating single-gait controllers, efficient and reliable transitioning between gaits has been a consistent challenge for legged robotics. Many robots use a heuristic controller that initiates a gait transition by either stopping locomotion entirely and then performing a sequence of procedures to guide the system into another gait pattern or adding energy into the system by providing a thrust during the stance phases. These existing controllers usually generate abrupt changes in center of mass trajectories or leg speeds (Hyun et al., 2014; Hyun et al., 2016). Most recently, reinforced learning controllers (Hwangbo et al., 2019; Siekmann et al., 2020) have been proposed to enable smooth and stable gait changes. However, this approach not only requires a large amount of data gathered from a particular application, but very limited knowledge can be learned about why and how this type of controller might outperform its conventional counterparts. Empirical data from animals has informed theoretical models to explain how gait transitions can be initiated across a broad range of speeds, potentially reveal new methodologies for synthesizing switching controllers.

For quadrupedal locomotion, gaits can be modeled as dynamical systems for which gaits with inter-limb coordination are stable attractors (Schöner et al., 1990). In these models, gait transitions associated with lack of coordination can be identified as bifurcations along gait system paths in parameter space. Genetic knockouts in pattern-generating neural pathways confirm that changes in synchronization between fore-hind and left-right leg pairs can induce a gait transition as speed increases (Danner et al., 2016). Breaking coordination between limbs has been successfully used as a mechanism to transition a quadrupedal robot from walking to trotting (Shinya et al., 2013). Previous studies have described how changes in gait features (i.e., leg contact angle, timing of gait events) result in gait transitions, it is difficult to translate these findings into robotic controllers but without understanding how model dynamics result in such changes in gait features. For bipedal locomotion, Geyer et al. (2006) found that a unified SLIP model can explain both bipedal walking and running gaits, which suggests that these two gaits are different oscillation modes of the same mechanical system with different energy levels. This insight has been useful for predicting gait transitions in cursorial bipeds (Gan et al., 2018b).

Here, we built upon previous template models (Geyer et al., 2006; O’Connor, 2009; Shen and Seipel, 2012) to provide the first insights into the factors determining the gait transitions of non-cursorial bipeds, such as jerboas. First, we experimentally measured Lesser Egyptian jerboa (*Jaculus*) kinematics and dynamics for each gait across a broad range of speeds. We used numerical optimization to match an extended SLIP model (Gan et al., 2018b) to the jerboa data. The resulting walking and running gaits were similar to the ones found in (Geyer et al., 2006). However, while the previous model required directly changing the angle of attack, the passive dynamics of the proposed model determine swing leg motion to generate different gaits. As a result, many other gaits, including those that require two different leg contact angles (e.g., asymmetrical bipedal skipping) emerge from the proposed model as a natural continuation from the gait search. We formally defined asymmetrical running, a jerboa gait that emerged from the numerical search. Using a detailed parameter scan, we identified two distinct mechanisms to induce a transition between these four gaits (walking, running, skipping and asymmetrical running). The proposed bipedal model that couples the neutral angle of both legs during the swing phase can change this angle to induce a gait transition. Alternatively, the model can uncouple the offset between the neutral angle of each leg during the swing phase to induce a gait transition. With these two mechanisms, the extended SLIP model is capable of matching the jerboa pattern of transitioning between gaits across a broad range of speeds. We also found that on the Poincaré section, the fixed points of the skipping gait are in close proximity to solutions found for all other gaits, which explains why jerboas transition to and from skipping gaits most frequently (Moore et al., 2017). Thus, this extended SLIP model matches empirical jerboa kinematics and dynamics, predicts gait transitions throughout a broad range of speeds, and provides a mechanism for initiating these gait transitions.

## 2 Methods

### 2.1 Animal Experiments

Details of the data collection procedure were reported in a previous publication in which the speeds and acceleration ranges associated with each gait were determined (Moore et al., 2017). Trials were collected from five captive male jerboas traveling along a narrow track (2 × 0.15 × 0.4 m^3^) over a two-axis force platform (0.06 × 0.12 m^2^) and past a high-speed video camera recording at 500 fps. We visually categorized the gait of each stride by footfall pattern. Both feet striking and lifting off simultaneously were considered hopping. Overlapping but non-simultaneous foot strikes were considered skipping, according to previous work (Moore et al., 2017). If the same leg maintained the leading foot position, this gait would be equivalent to a bipedal gallop, as defined in previous gait research (Schropfer et al., 1985; Gan et al., 2018b). An aerial phase between each foot strike was considered running if each aerial phase was approximately the same duration.

**FIGURE 1:**
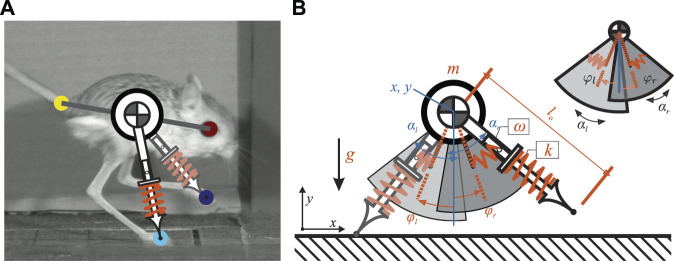
**(A)** shows how the proposed model relates to a jerboa. The COM location is approximated as the midpoint between the eye and the tail-base. The leg angles are estimated by the orientation of the line segments connecting the COM to the feet **(B)** illustrates the proposed SLIP model with passive swing leg motion. There are four continuous states (shown in blue) including the position of the torso (*x*, *y*) as well as the leg angles $\left(\alpha_{l_{0}},\alpha_{r_{0}}\right)$ . Model parameters are highlighted in red, including total body mass, *m*, uncompressed leg length, *l*_*o*_, gravity, *g*, and leg stiffness, *k*. Adding a torsional spring to a SLIP model enables motion of passive swing leg. The rotational speeds of both swing legs are determined by *ω* and the neutral leg angle are *φ*_*l*_ and *φ*_*r*_ respectfully. Note that the neutral leg swing angle for the right leg, *φ*_*r*_, is different from that of the left leg, demonstrating an uncoupled model. A simplified version of the model showing the range of the swing leg motion is also shown in the top-right corner.

To extract the kinematic data (i.e., center of mass (COM) locations and leg angles over one stride) from the video recordings, we used **DeepLabCut**, a markerless pose estimation framework leveraging a deep neural network (DNN) (Mathis et al., 2018). In this study, 35 videos that contained a whole stride of a single gait pattern were used to train the DNN. All three common jerboa gaits reported in (Moore et al., 2017) (i.e., hopping, skipping, running) were included in this study. Roughly 1/3 of the total frames of each video were selected as the training data set. In these frames, we manually labelled the location of the eye, the tail-base, and the two feet, as shown in Figure 1 A. We estimated the COM location as the midpoint between the eye and the tail-base. Then the leg angles were calculated as the orientation of the line segments connecting the COM to the feet.

### 2.2 Model Description

The proposed model used in this study consists of a point mass as the main body, with mass *m*, and two massless legs, as illustrated in Figure 1B. The vertical and horizontal positions of the main body were defined by the variables *x*(*t*) and *y*(*t*), respectively. Left and right legs (with index $i\in \left[l,r\right]$ ) were modeled as massless linear springs with resting leg length *l*_*o*_ and total spring stiffness *k*. Both legs were connected to the main body through frictionless rotational joints, with the joint angle *α*_*i*_(*t*) measured from the vertical axis (positive in the counterclockwise direction). Comparing with the convectional SLIP model, which ignores swing leg motions by setting the leg to predefined angles of attack immediately after lifting off, we added a torsional passive spring to control the leg swing motion during the flight phase of each leg. This is similar to the monopedal SLIP model with hip torque and leg damping proposed by (Shen and Seipel, 2012), in which active constant hip torques and leg dampings during the stance phase improved the stability and robustness of locomotion. In contrast, the torsional springs in our model provide passive torques enable the rotational motions of the swing legs to facilitate gait transitions. The torsional spring directly connected the leg to the main body at angle, *φ*_*i*_ (hereafter referred to as the neutral swing leg angle (NSLA), measured with respect to the vertical direction (Figure 1B). By fixing the oscillation frequency *ω*, this torsional spring dictates the swing leg rotational speed and amplitude and determines the desired contact angle at the moment of touchdown. Because we can assume that the torsional spring stiffness and the foot mass have infinitesimal values, they do not affect stance leg kinematics or dynamics (Gan et al., 2018b).

**FIGURE 2:**
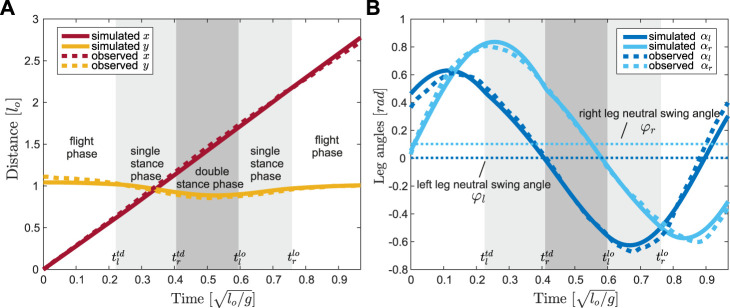
Trajectory optimization results, in solid lines, for **(A)** the COM position $\left[x,y\right]$ and **(B)** the leg angles $\left[\alpha_{l_{0}},\alpha_{r_{0}}\right]$ of trial 1802− *j*30 closely match the empirical data, in dashed lines. The fitting result shows whole stride, starting from the apex transition, when $t\in \left[0,T\right]$ in the flight phase (white background). The single stance phases are indicated by the lighter gray background and the double stance phases are indicated by the darker gray background. Compared to the rotational motion of the left leg (dark blue), the right leg rotations are translated anteriorly (light blue), which is reflected in our model by setting the neutral leg swing angles to *φ*_*l*_ =0.032 rad and *φ*_*r*_ =0.137 rad, marked by horizontal dotted lines in **(B)**. The difference between *φ*_*l*_ and *φ*_*r*_ is the offset in uncoupled leg models.

In our work, we ran optimizations to fit the trajectories of leg angles (Figure 2) to determine the oscillation frequency *ω*. The full set of parameters of the proposed model is denoted as ${\vec{p}}^{T}:=\left[m,l_{o},g,k,\omega ,\varphi_{l},\varphi_{r}\right]$ .

The total stride time was defined as *T* and its value was not known before finding a gait pattern of the proposed model. Without loss of generality, we chose the apex transition $\left({\dot{y}}_{o}=\dot{y}\left(T\right)=0\;\sqrt{l_{o}g}\right)$ as the Poincaré section. This means that the beginning of each gait cycle was defined as the peak of the aerial phase, when the COM was highest off of the ground. To reproduce all observed bipedal gait patterns of jerboas and analyze their transitions, we did not prescribe a specific footfall pattern. Instead, we introduced four timing variables $t_{i}^{j}$ (with index $i\in \left[l,r\right]$ , $j\in \left[td,lo\right]$ ), for the touchdown and liftoff events that are confined within the time interval of one stride $\left[0,T\right)$ . Their values were determined and sorted through the gait finding process, as detailed in Section 2.4. The full set of timing variables of the proposed model is encapsulated in a vector ${\vec{t}}^{T}:=\left[t_{l}^{td},t_{l}^{lo},t_{r}^{td},t_{r}^{lo},T\right]$ .

### 2.3 Equations of Motion

Using the position and velocity vectors ${\vec{q}}^{T}:=\left[x,y,\alpha_{l},\alpha_{r}\right]$ , ${\vec{\dot{q}}}^{T}:=\left[\dot{x},\dot{y},{\dot{\alpha }}_{l},{\dot{\alpha }}_{r}\right]$ to describe the state of the system, we expressed the dynamics as a set of second-order time-varying differential equations $\vec{\ddot{q}}=f\left(\vec{q},\vec{\dot{q}},\vec{t},\vec{p}\right)$ that is parameterized by $\vec{p}$ . The equations of motion (EOM) were defined for the main body as:

$$\ddot{x}=F_{x}/m,\;\ddot{y}=F_{y}/m-g,$$ (1)

where *F*_*x*_ and *F*_*y*_ represent the net forces and torques generated by the leg pairs. The dynamics of the leg pairs depended on whether the legs were in contact with the ground. During the swing phase, the leg was set to its uncompressed original length *l*_*o*_ and the leg angular accelerations were defined by:

$${\ddot{\alpha }}_{\text{swing},i}=1/l_{o}\left(\ddot{x}\cos\alpha_{i}+\left(g+\ddot{y}\right)\sin\alpha_{i}\right)+\omega^{2}m\left(\alpha_{i}-\varphi_{i}\right),$$ (2)

During stance, we assumed that the ground has infinite friction so that the stance foot did not slide on the ground. A holonomic constraint was introduced to make sure the horizontal position of the contact foot (*x*_c,*i*_) was stationary.

$$x_{\text{c},i}-x-y\tan\alpha_{i}=0,$$ (3)

Whenever a leg entered stance phase, the angular acceleration of that leg was determined by the accelerations of the main body, which was directly computed from the above ground constraint by taking the time derivative twice:

$${\ddot{\alpha }}_{\text{stance},i}=-2\;{\dot{\alpha_{i}}}^{2}\;\tan\left(\alpha_{i}\right)-\frac{2\;\dot{\alpha_{i}}\;\dot{y}}{y}-\frac{\ddot{x}+\ddot{y}\;\tan\left(\alpha_{i}\right)}{y\;\sec^{2}\left(\alpha_{i}\right)}.$$ (4)

In addition, at the moments of touch-down $t_{i}^{td}$ , the leg velocities were reset according to the holonomic constraint Equation (3), resulting in additional discrete dynamics to ensure zero stance foot velocity when integrating Equation (4). $t_{i}^{td+}$ and $t_{i}^{td-}$ were used to indicate the moments right after and before the touch-down event of a leg, respectively.

$$\vec{\dot{q}}\left(t_{i}^{td+}\right)=h\left(\vec{q}\left(t_{i}^{td-}\right),\vec{\dot{q}}\left(t_{i}^{td-}\right)\right).$$ (5)

Posterior neutral leg swing angles usually induced a premature touchdown event during anterior swing leg motion, causing the leg to immediately rotate posteriorly and inducing a large angular velocity reset (Eq. (5)). Because this behavior is rarely seen in jerboa locomotion, we terminated the numerical search when this phenomenon was detected.

### 2.4 Gait Finding and Continuation

Due to the nonlinearity and the hybrid nature of the EOM presented in Section 2.3, it was not possible to find explicit periodic solutions of the proposed model. Therefore, in this work we identified gait patterns as numerical solutions dictated by the initial condition of the continuous states ${\vec{q}}_{o}$ , ${\vec{\dot{q}}}_{o}$ and system parameters ${\vec{p}}^{T}$ . Because a gait of the system is a periodic motion, finding a bipedal gait in this model was equivalent to solving a root of the following set of constraint equations:

$$\begin{array}{ccc} & & \text{find}\;{\vec{q}}_{o},{\vec{\dot{q}}}_{o},\vec{t}\\ & & \text{such that:}\\ & & \left\{\begin{array}{c} \vec{q}\left(T\right)-{\vec{q}}_{o}=0,\;\\ \vec{\dot{q}}\left(T\right)-{\vec{\dot{q}}}_{o}=0,y\left(t_{i}^{j}\right)-l_{o}\cos\left(\alpha_{i}\left(t_{i}^{j}\right)\right)=0\;\text{for every}\;i\in \left[l,r\right],j\in \left[td,lo\right].\; \end{array}\right. \end{array}$$ (6)

This is a passive model with no additional controllers or actuators. When the parameters of the proposed model were fixed, there were 13 variables $\left({\vec{q}}_{o},{\vec{\dot{q}}}_{o},\vec{t}\right)$ and 12 constraints (equalities listed in Eq. (6)). For such a conservative model, the total energy stored in the system can be calculated as $E=\frac{1}{2}m{\dot{x}}_{o}^{2}+\frac{1}{2}m{\dot{y}}_{o}^{2}+mgy_{o}$ , so varying the initial conditions is equivalent to changing the total energy. As a result, periodic solutions formed one-dimensional manifolds (hereafter referred to as **branches**) as the total energy stored in the system varied. We integrated the system over a complete stride using the Runge-Kutta-Fehlberg Method (RKF) (Fehlberg, 1969) and solved for roots of the above equalities using the fsolve function of Matlab. Finding the first periodic motion (gait) of the proposed model requires a good estimation of the initial states. It is the easiest to start with a solution of zero forward speed in which the horizontal position of COM, the leg angles, and leg angular velocities remain at zeros during the whole stride. Once one periodic motion was found, we ran numerical continuations using the predictor and corrector method (Gan et al., 2018a) to quickly explore the adjacent periodic solutions and their transitions to other gait patterns. Because most of the gait transitions appeared from the numerical search as a **bifurcation point**, at which one of the Floquet multipliers of the system is equal to +1, the corresponding eigenvector was approximately directed towards the solution with the new gait pattern (Gan et al., 2018b).

In nature, jerboas move with step-to-step changes in stride length, direction, gait, and speed and rarely demonstrate exact periodic gait patterns. In this work, we assume they are utilizing a stabilizing controller for a desired limit cycle, which is changed discretely each step. We also assume that the state of the jerboa is always within the region of attraction of the controller and the desired limit cycles. Additionally, we only explored gaits with a left-leg phase advance because the motions of the left-advanced gaits and right-advanced gaits were identical when the leg parameters were the same and the two legs were switched. Thus, although they occurred in the animals, we did not mathematically explore gait transitions between left-advanced and right-advanced skipping gaits.

### 2.5 Parameter Identification

To reduce the number of free parameters and identify their values in the proposed model, we normalized all values in the model in terms of the total mass of the system, *m*, the uncompressed leg length, *l*_*o*_, and the gravity on Earth, *g* (Hof, 1996). The estimation of leg stiffness was based on the assumptions that legs were massless and that they behaved as simple linear springs. The period of the oscillation around the leg was therefore dictated by the spring stiffness, according to $\sqrt{k/m}$ . To estimate the swing leg oscillation frequency *ω*, and to determine how well the proposed model can explain the empirical motions of jerboas, we proposed the following optimization framework.

By solving Equation (6), the simulated model trajectories of positions and velocities of a periodic solution can be represented by a 3-tuple $\vec{X}:=\left({\vec{q}}^{⋆},{\vec{\dot{q}}}^{⋆},{\vec{t}}^{⋆}\right)$ . For the *n*th experimental trial, the residual function $C_{n}\left(\vec{X},\vec{p}\right)$ quantifies how well the model with a specific parameter set $\vec{p}$ predicted the kinematics of the locomotion pattern in jerboas. The empirical positions and velocities of jerboas from the *n*th experimental trial were denoted by ${\vec{q}}_{n}^{e}$ and ${\vec{\dot{q}}}_{n}^{e}$ , respectively. The value of this cost function was minimized as a nonlinear optimization problem with an optimal set of parameters, $\vec{p}$ :

$$C_{opt}=\underset{X,\;p}{\min}\left\{C_{n}:=\int_{0}^{T^{⋆}}{\left\|{\vec{q}}^{⋆}\left(t,p\right)-{\vec{q}}_{n}^{e}\left(t\right)\right\|}^{2}\;+{\left\|{\vec{\dot{q}}}^{⋆}\left(t,p\right)-{\vec{\dot{q}}}_{n}^{e}\left(t\right)\right\|}^{2}\;dt\right\}.$$ (7)

This algorithm was implemented in Matlab using sequential quadratic programming (SQP). Each optimization problem can be solved on a regular desktop computer with an Intel Core i7 3.4 GHz processor in a few minutes.

## 3 Results

In this study we created a high-fidelity template model that can accurately reproduce jerboa gait transitions. First we demonstrate a simulated skipping gait pattern from the template model using the proposed optimization algorithm. Next, Section 3.2 formally defines the symmetric and asymmetric jerboa gaits, including the first description of the asymmetrical running gait. Then, we analyze the effects of varying NLSA in two different scenarios. In Section 3.3, the NLSA of both legs are varied together and thereafter referred as the coupled leg model. In Section 3.4, we allow offset, or differences, in the right and left NLSA and call it the uncoupled leg model. In the last section, we validate our model by comparing our predictions to empirical gait transition data from jerboas. The framework we created and a video showing the jerboa gait transitions have been included in the Supplementary Material.

### 3.1 Optimized Model Parameters Recreate Empirical Observations

As mentioned in the previous sections, the full set of parameters of the proposed model was denoted as ${\vec{p}}^{T}:=\left[m,l_{o},g,k,\omega ,\varphi_{l},\varphi_{r}\right]$ . All values were normalized and *m*, the uncompressed leg length, *l*_*o*_, and the gravity on Earth, *g* which were all set to a value of one. Based on the methods in Section 2.5, the mean value and the standard deviation of the leg spring stiffness was estimated at *k* = 19.24 ± 2.43 *mg*/*l*_*o*_. Swing leg oscillation frequency *ω* varied minimally across trials for each jerboa (e.g., $6.77\pm 0.18\;\sqrt{g/l_{o}}$ for j30, $5.75\pm 0.65\;\sqrt{g/l_{o}}$ for j38). Because the deviations of both leg stiffness and swing leg oscillation frequency were relatively small in our entire data set, we assumed they were not the major contributors to the gait transitions in jerboas. Thus, we set leg stiffness to 20 *mg*/*l*_*o*_ for the subsequent simulations and used $6.5\;\sqrt{g/l_{o}}$ . For a given set of parameters, we exhaustively searched for solutions, which resulted in a maximum forward speed of $29\sqrt{gl_{o}}$ . Although these branches included unrealistic speeds, all gait transitions emerged below $8\sqrt{gl_{o}}$ . The optimized parameters (Table 1) produced trajectories that closely match the empirical jerboa COM location and both leg angles (coefficient of determination 0.83 < R-squared < 0.99, Figure 2).

**TABLE 1:** Optimized initial states and system parameters of the proposed model associated with all 12 empirical trials of jerboa skipping locomotion are listed in this table. The optimized trajectory of trial 1802-j30 corresponds to Figure 2. All states and parameters are normalized with respect to the total mass of the system, *m*, the uncompressed leg length, *l*_*o*_, and the gravity on Earth, *g*.

Jerboa	j30				j38				j44			j61
Recording	1802	1826	1827	1828	2007	2018	2029	2035	1138	1317	1320	1940
States	sim exp	sim exp	sim exp	sim exp	sim exp	sim exp	sim exp	sim exp	sim exp	sim exp	sim exp	sim exp
${\dot{x}}_{o}$ $\sqrt{gl_{o}}$	2.86 2.22	4.27 4.05	5.01 5.47	4.07 3.61	3.88 4.73	3.83 4.06	4.64 5.09	3.64 3.52	1.79 1.53	1.82 1.99	2.08 2.30	2.15 2.13
*y*_*o*_ *l*_*o*_	1.04 1.11	0.96 1.01	0.90 0.96	0.86 0.88	1.15 1.26	1.16 1.22	0.98 1.09	1.08 1.11	1.33 1.36	1.19 1.34	1.07 1.17	1.10 1.15
${\dot{y}}_{o}$ $\sqrt{gl_{o}}$	0.01 0.15	0.01–0.21	0.00–0.04	0.00–0.26	-0.12–0.16	0.00 0.03	-0.07–0.91	-0.09 0.07	-0.18 0.12	-0.02–0.33	0.00–0.10	0.00–0.82
$\alpha_{l_{0}}$ *rad*	0.35 0.36	0.27 0.30	0.55 0.55	0.15 0.06	0.36 0.47	-0.17–0.64	0.17 0.08	0.19 0.13	-0.32–0.38	0.01–0.06	-0.15–0.36	0.23 0.24
$\dot{\alpha_{l_{0}}}$ $\sqrt{g/l_{o}}$	3.18 3.90	5.18 7.72	4.07 4.33	5.14 7.41	3.61 2.28	3.21 4.39	5.16 8.12	4.47 4.72	2.52 2.87	1.84 2.20	1.79 2.89	2.33 1.27
$\alpha_{r_{0}}$ *rad*	0.12 0.03	0.66 0.76	0.68 0.69	0.45 0.33	0.36 0.32	-0.24–0.32	-0.08–0.29	-0.21–0.45	0.56 0.29	0.41 0.44	0.49 0.41	-0.30–0.33
$\dot{\alpha_{r_{0}}}$ $\sqrt{g/l_{o}}$	4.15 7.09	3.59 1.92	2.21 1.33	4.49 7.79	2.83 2.80	4.61 5.52	5.66 6.89	4.33 5.52	-0.21 1.56	0.91 0.89	1.67 3.11	3.33 2.28
**Timing**	**sim exp**	**sim exp**	**sim exp**	**sim exp**	**sim exp**	**sim exp**	**sim exp**	**sim exp**	**sim exp**	**sim exp**	**sim exp**	**sim exp**
$t_{l}^{td}$ $\sqrt{l_{o}/g}$	0.45 0.32	0.44 0.50	0.53 0.49	0.49 0.54	0.47 0.43	0.38 0.36	0.30 0.28	0.48 0.47	0.43 0.51	0.45 0.50	0.52 0.52	0.27 0.31
$t_{l}^{lo}$ $\sqrt{l_{o}/g}$	0.60 0.56	0.63 0.68	0.68 0.67	0.61 0.74	0.59 0.60	0.52 0.56	0.47 0.44	0.64 0.66	0.68 0.78	0.65 0.71	0.65 0.74	0.68 0.66
$t_{r}^{td}$ $\sqrt{l_{o}/g}$	0.45 0.45	0.29 0.36	0.29 0.33	0.46 0.43	0.64 0.60	0.48 0.47	0.43 0.48	0.61 0.61	0.44 0.31	0.26 0.29	0.37 0.30	0.60 0.49
$t_{r}^{lo}$ $\sqrt{l_{o}/g}$	0.81 0.68	0.49 0.57	0.56 0.51	0.62 0.61	0.72 0.78	0.60 0.65	0.62 0.69	0.85 0.79	0.57 0.50	0.47 0.46	0.51 0.52	0.85 0.77
Duty Factor	0.26 0.24	0.19 0.20	0.21 0.18	0.14 0.19	0.12 0.17	0.13 0.19	0.18 0.19	0.20 0.18	0.19 0.23	0.21 0.19	0.14 0.22	0.33 0.31
Stride Time $\sqrt{l_{o}/g}$	0.93 0.97	0.91 0.79	0.68 0.69	0.84 0.81	0.91 0.90	1.27 1.24	0.90 0.88	0.90 0.92	1.77 1.70	1.66 1.60	1.51 1.44	0.83 0.87
Stride Length *l*_*o*_	2.69 2.73	3.89 3.30	3.38 3.34	3.37 3.13	3.52 3.38	5.01 4.84	4.17 4.11	3.28 3.40	3.05 2.97	3.13 2.93	3.18 2.88	1.91 2.01
Average Speed $\sqrt{gl_{o}}$	2.88 2.81	4.26 4.13	4.99 4.85	4.00 3.83	3.86 3.76	3.99 3.88	4.64 4.70	3.64 3.71	1.73 1.75	1.91 1.83	2.12 1.99	2.32 2.44
**R-squared**												
*X*	0.99	0.99	0.99	0.99	0.99	0.99	0.99	0.99	0.99	0.99	0.98	0.99
*Y*	0.88	0.97	0.90	0.94	0.83	0.92	0.84	0.93	0.95	0.89	0.88	0.95
*α*_*l*_	0.99	0.98	0.99	0.99	0.99	0.90	0.99	0.99	0.99	0.97	0.86	0.97
*α*_*r*_	0.99	0.99	0.99	0.99	0.99	0.99	0.98	0.98	0.97	0.99	0.97	0.98
**Parameters**												
*ω* $\sqrt{g/l_{o}}$	6.49	6.87	6.99	6.75	5.86	4.84	6.37	5.94	3.27	3.63	4.08	7.17
*φ*_*l*_ *rad*	0.02	0.23	0.32	0.28	0.03	-0.17	-0.45	-0.12	-0.15	0.18	0.26	-0.10
*φ*_*r*_ *rad*	0.17	0.03	0.00	0.22	0.40	-0.01	-0.09	0.07	0.16	-0.10	-0.04	-0.05

### 3.2 Symmetrical and Asymmetrical Gaits Lie on Two Distinct Continua

**FIGURE 3:**
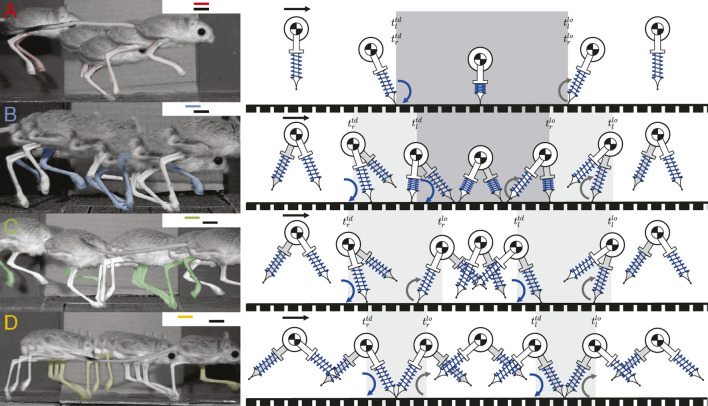
The apex transitions, touchdowns, and liftoffs for one stride of four different gait patterns are demonstrated by jerboas on the left, with inset gait diagrams showing footfall patterns, the corresponding simulated gait patterns using our model are shown on the right. The right leg of jerboa is shown in white and the left leg is in the same color as the corresponding gait branches shown in the inset gait diagram and in Figure 4. The left leg of the model is shown in grey and the right leg is in white **(A)** shows hopping in which both feet strike and lift off simultaneously **(B)** shows skipping with overlapping but non-simultaneous foot strikes (**C**) shows asymmetrical running with two different aerial phases **(D)** shows symmetrical running which contains two aerial phases with approximately the same duration. Blue curved arrows indicate leg touchdown $\left(t_{i}^{td}\right)$ and the gray curved arrows denote liftoff events $\left(t_{i}^{lo}\right)$ .

Based on the numerical search described in Section 2.4, we found periodic solutions for five different gait patterns: walking, hopping, skipping, symmetrical running, and asymmetrical running (Figure 3). The definitions of the first four gait patterns follow the conventions described in Section 2.1 and in previous research (Happold, 1967; Eilam and Shefer, 1997; Moore et al., 2017), while asymmetrical running is a novel gait presented this study (Section 3.2.2).

#### 3.2.1 The Nominal Model has Neutral Leg Swing Angles of Zero

**FIGURE 4:**
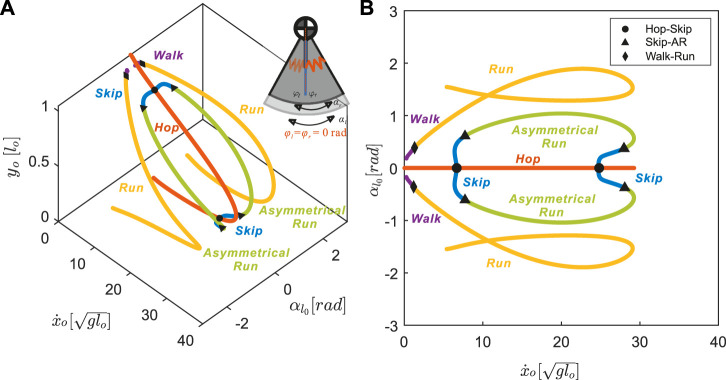
The nominal model with neutral swing leg angles of zero, *φ*_*l*_ = *φ*_*r*_ =0[*rad*], results in symmetrical gait structures (all other parameters were fixed as described in Section 3.1). Each point on the branches represents a distinct periodic motion, or a stationary point on **(A)** a 3D projection and **(B)** a 2D projection of the Poincaré section $\left(\dot{y}=0\right)$ with respect to the apex height *y*_*o*_, forward speed ${\dot{x}}_{o}$ , and left leg angle $\alpha_{l_{0}}$ . Hopping to skipping, skipping to asymmetrical running, and walking to symmetrical running transition are represented by circles, triangles, and diamonds respectively. For gait solutions with a negative leg angle, the opposite leg has a phase advance.

All the identified locomotion patterns form one-dimensional branches connected to one another through bifurcation points on the Poincaré section (Figure 4). These solution branches are hereafter referred to as the **gait structure**. Just as in our previous SLIP model (Gan et al., 2018a), setting both the neutral leg swing angles to zero results in a nominal gait structure symmetric about the plane $\alpha_{l_{0}}=0$ rad.

Symmetrical gaits, walking and running, form one continuum (purple and yellow in Figure 4). For symmetrical gaits, identical leg movements are out of phase by half a stride $\left(|t_{l}^{j}-t_{r}^{j}|=T/2\right)$ (Hildebrand, 1967). Walking (purple in Figure 4) appears only at low speeds and is characterized by a lack of aerial phase (i.e., $0<t_{r}^{td}<t_{l}^{lo}<t_{l}^{td}<t_{r}^{lo}<T$ ). When the forward speed reaches ${\dot{x}}_{o}=1.21\sqrt{gl_{o}}$ (diamonds in Figure 4), one leg strikes the ground at the exact moment when the other leg leaves the ground, i.e. $t_{i}^{td}=t_{\bar{i}}^{lo}$ where $i\in \left[l,r\right]$ and $\bar{i}$ denotes the index of the opposite leg. As speed further increases, liftoff of one foot occurs before touchdown of the other foot and walking smoothly transitions to running with aerial phases between each footfall, i.e. $0<t_{r}^{td}<t_{r}^{lo}<t_{l}^{td}<t_{l}^{lo}<T$ (Figure 3D, yellow in Figure 4).

A distinct continuum connects the three **asymmetrical gaits**: hopping, skipping, and asymmetrical running (red, blue, and green lines in Figure 4), for which the phase shift between legs is not equal to half a stride $\left(|t_{l}^{j}-t_{r}^{j}|\neq T/2\right)$ (Hildebrand, 1977). Along the hopping branch (Figure 3A, red in Figure 4), leg motions are synchronized, i.e. $0<t_{r}^{td}=t_{l}^{td}<t_{r}^{lo}=t_{l}^{lo}<T$ . This synchronization is broken, via hopf bifurcations (Hassard et al., 1981, Chapter 1), at two different speeds (circles in Figure 4B), both leading to skipping (Figure 3B, blue in Figure 4B) with overlapping footfall patterns (i.e. $0<t_{r}^{td}<t_{l}^{td}<t_{r}^{lo}<t_{l}^{lo}<T$ ).

#### 3.2.2 Definition of Asymmetrical Running

At skipping speeds ${\dot{x}}_{o}=7.72\sqrt{gl_{o}}\text{or }28.06\sqrt{gl_{o}}$ (triangles in Figure 4), previously overlapping touchdown and liftoff events occur simultaneously (e.g., $t_{l}^{td}=t_{r}^{lo}$ ). At intermediate speeds, a short aerial phase emerges in the middle of the stride. As opposed to symmetrical running, gaits in which two aerial phases are unequal in duration (Figure 3C) are **asymmetrical running**, with distinct contact angles for each leg and footfall pattern $0<t_{r}^{td}<t_{r}^{lo}<t_{l}^{td}<t_{l}^{lo}<T$ . In the following sections we will show that the asymmetrical running gait plays an important role in gait transitions and appears ubiquitously in the gait structure as we sweep the parameter space spanned by neutral leg swing angles.

### 3.3 Coupled Changes in Neutral Leg Swing Angle Shift the Speeds of Existing Gait Transitions

#### 3.3.1 Anterior Shifts in Coupled Neutral Leg Swing Angle Preserve Symmetrical Gait Structure

**FIGURE 5:**
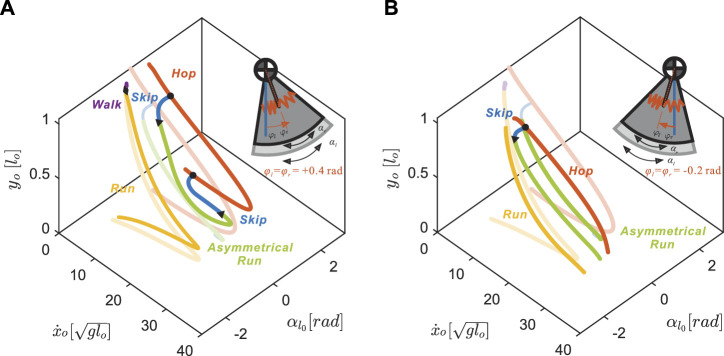
Coupled changes in the neutral leg swing angles affect the gait structure of the nominal model (transparent lines) on the Poincaré section with respect to the apex height, *y*_*o*_, forward speed, ${\dot{x}}_{o}$ , and left leg angle, $\alpha_{l_{0}}$ **(A)** A positive neutral leg swing angle, *φ*_*l*_ = *φ*_*r*_ =0.4 rad, preserves and translates gait structure **(B)** A negative neutral leg swing angle, *φ*_*l*_ = *φ*_*r*_ =−0.2 rad, alters gait structure. All other parameters including leg stiffness, *k*, and swing leg oscillation frequency, *ω*, were fixed as described in Section 3.1. In both plots, only gaits with left-leg-advanced are shown because symmetry is preserved. The inset model diagrams show the range of leg rotational motions (dark grey sector for $\alpha_{r_{0}}$ , light grey sector for $\alpha_{l_{0}}$ ) and the neutral leg angles.

**FIGURE 6:**
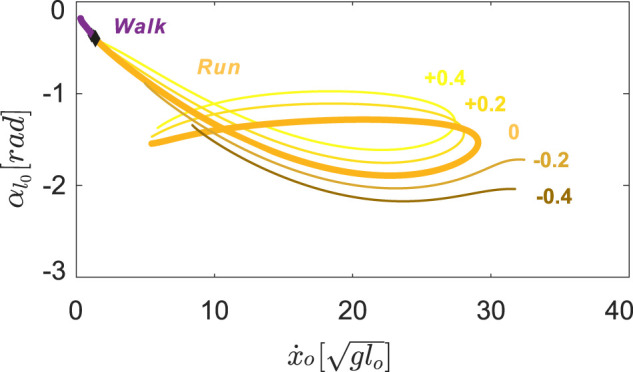
Symmetrical gait structures for $\varphi_{l}=\varphi_{r}\in \left[-0.4,-0.2,0,+0.2,+0.4\right]$ rad. Positive neutral leg swing angles retain the running branch shape and transition speed. Negative neutral leg swing angles shrink the running gait structure towards the mid-speed region, eliminating walking.

As we increased the values of *φ*_*l*_ = *φ*_*r*_, the legs immediately rotated anteriorly at liftoff (see the inset at the top right corner of Figure 5A). After reaching the maximum anterior position, the legs would rotate posteriorly prior to ground contact, i.e. swing leg retraction (Seyfarth et al., 2003). Despite this change in kinematics, the transitions to walking (diamonds in Figure 6) remained approximately at the same speed, $1.2\;\sqrt{gl_{o}}$ .

On the other hand, negative neutral leg swing angles (posteriorly shifted) induced changes in the shape of the gait branches. As shown in Figure 5B, at low speeds, there were no viable solutions for walking or running because the swing legs failed to maintain a positive leg angle at the moment of touch-down, which is required to keep moving in the positive horizontal direction.

At higher speeds, the curved regions of the branches, corresponding to solutions that include swing leg retraction, disappeared. Instead, higher speed running solutions involved swing legs rotating forward at the moment of touch-down, which induced an angular velocity reset and large plastic collision losses. When we further decreased the value of neutral leg swing angles, the entire running branch shrank towards the mid-speed region, eventually vanishing at approximately *φ*_*i*_ = −0.8 rad.

#### 3.3.2 As Coupled NLSA Varies, the Speed of Higher Speed Transitions Changes More Than Lower Speed Transitions

**FIGURE 7:**
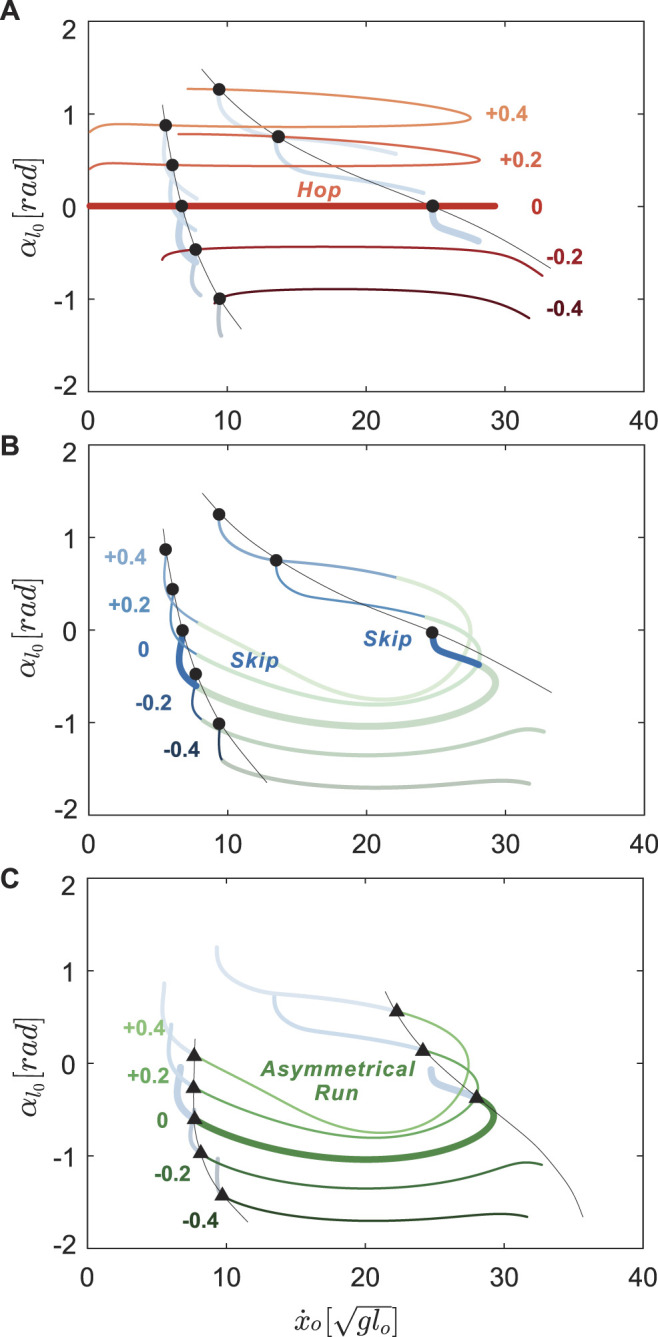
Hopping (A), skipping **(B)**, and asymmetrical running gait branches (C) with $\varphi_{l}=\varphi_{r}\in \left[-0.4,-0.2,0,+0.2,+0.4\right]$ rad. Thicker colored lines represent the nominal gait structure (Figure 4). Hop-skip transitions are circles in **(A)** and **(B)**, skip-running transitions are triangles in **(C)**. Thin black lines trace gait transitions across varying neutral leg swing angles. The higher speed hop - skip transition point showed more variation in speed with changes in NLSA than the lower speed transition point.

Hopping solutions were found in the range of −0.8 < *φ*_*i*_ < 1.5 rad (Figure 7A). Minor changes in the shape of hopping branches were observed as we varied *φ*_*i*_ in the positive direction. However, as we gradually decreased the values of the NLSA, periodic hopping gaits were only identified at mid-speed ranges with reduced landing impact. As in running gaits, hopping with emergent swing leg retractions were identified only at moderate speeds.

For all hopping branches with different NLSA values (red curves in Figure 7A), there was always at least one hop - skip transition point (circles in Figure 7A,B) and no transitions to asymmetrical running. As the hopping branch crosses a bifurcation point, the symmetry in the leg motions is broken, desynchronizing motions of the leg pair to generate skipping gaits with a staggered timings of touchdown events (see Figure 3A,B). One hop - skip transition usually occurred at lower speeds and another at higher speeds, near the turning points. The location of the low speed hop - skip transition point varied minimally as the NLSA were altered. In contrast, the higher speed hop - skip transition point showed more variation in speed with changes in NLSA than the lower speed transition point (Figure 7A). For negative *φ*_*i*_, the swing leg angular velocity reset occurred before the high speed transition points could be found.

Starting from the hop - skip transitions points (circles), skipping gaits bifurcated from the hopping branches and emerged at discontinuous locations on the Poincaré section (Figure 7B). The lower speed branch was shorter than the higher speed branches for positive neutral leg swing angles. The branches with higher average forward speeds disappeared very quickly because of the impractical swing leg behavior with a maximal forward speed around $29\sqrt{gl_{o}}$ . The asymmetrical running gait provided a smooth transition branch that bridged the two isolated skipping branches for the same neutral leg swing angle (Figure 7C).

Skipping solutions were found in the range of −0.7 < *φ*_*i*_ < 1.0 rad. However, when the NLSA were larger than +0.4 rad, the maximum value during swing motion of the legs exceeded a value of 1.7 rad (*π*/2), which would be biologically unrealistic. Therefore, only results from $\varphi_{l}=\varphi_{r}\in \left[-0.4,+0.4\right]$ are shown. The skip - asymmetrical run transitions showed a similar pattern to the hop - skip transitions. The lower speed skip - asymmetrical run transitions always occurred when the forward speed reached approximately $8\;\sqrt{gl_{o}}$ . For the higher speed transitions, the locations varied more with positive changes in neutral leg swing angle. As soon as the neutral leg swing angles became negative, higher speed transitions between skipping and asymmetrical running were no longer viable.

### 3.4 Uncoupled Changes in Neutral Leg Swing Angle Introduce New Transitions

**FIGURE 8:**
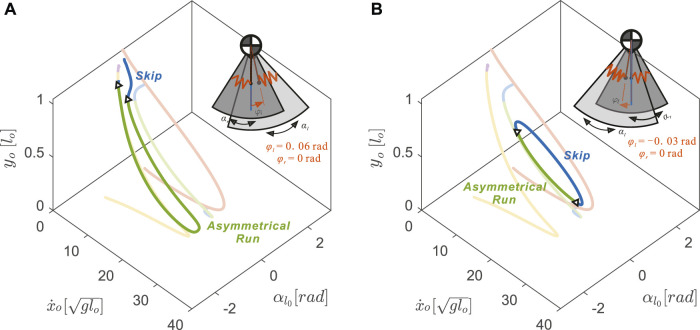
Uncoupling the neutral leg swing angles (*φ*_*l*_ ≠ *φ*_*r*_) resulted in drastic changes in 3D gait branch shape with respect to the nominal model (transparent curves from Figure 4) **(A)** An anterior shift, *φ*_*l*_ =+0.06 rad, caused the asymmetrical running branch to subsume portions of the previously symmetrical running branch (yellow) **(B)** A posterior shift, *φ*_*l*_ =−0.03 rad, caused the skipping branch to subsume portions of the previously symmetrical hopping gait (red) to form a closed loop. The inset model diagrams show the range of leg rotational motions (dark grey sector for $\alpha_{r_{0}}$ , light grey sector for $\alpha_{l_{0}}$ ) and the neutral leg angles.

Uncoupling the neutral swing angles for each leg, i.e. *φ*_*l*_ ≠ *φ*_*r*_, resulted in drastic changes in both gait structure and the locations of gait transitions (Figure 8). Without symmetry, skipping and asymmetrical running became the only two feasible gait patterns. Furthermore, the model symmetry between left-leg-advanced and right-leg-advanced solutions were no longer preserved for more offset values, |*φ*_*l*_ − *φ*_*r*_| > 0, of the uncoupled model because simply switching the leg angles would not result in identical COM motion. For clarity, only the left-leg-advanced solutions for small offset values were included in the analysis.

With uncoupled neutral leg swing angles, more skipping and asymmetrical running gait solutions became possible by slightly disrupting the symmetry of the symmetrical running and hopping gaits. With positive offset in the left neutral leg swing angle (*φ*_*l*_ − *φ*_*r*_ > 0), the asymmetrical running branch elongated by closely matching the symmetrical running gait (see Figure 8A). In contrast to the skipping branch of the coupled leg model, which connected directly to the hopping branch (opaque blue curve in Figure 8A), the uncoupled skipping branch continued to the lower speed regions in which the flight phases became shorter and shorter until they were replaced by a double stance phase.

On the other hand, with negative offset, the skipping branch (blue curve in Figure 8B) higher speed regions closely resemble the symmetrical hopping gait (red curve). When speeds were too fast or too slow, these skipping gaits joined with the asymmetrical running branch and formed the 1-dimensional manifold as a closed loop. The size of this loop decreased with the value of the left neutral leg angle. No solutions were found past *φ*_*l*_ = −0.14 rad, where the solution branch became a single dot.

**FIGURE 9:**
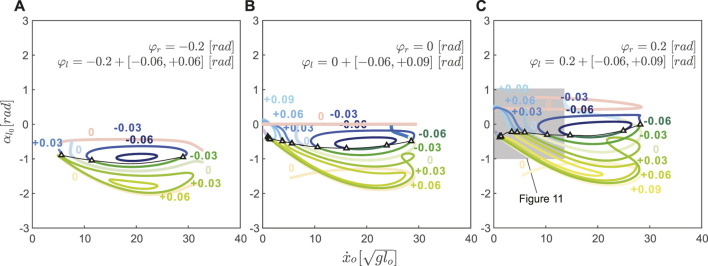
The fixed neutral leg swing angle, *φ*_*r*_, and the varying neutral leg swing angle, *φ*_*l*_, interact to affect asymmetrical gait structure in uncoupled models. In all plots, the coupled $\varphi_{l}=\varphi_{r}\in \left[-0.2,0,+0.2\right]$ rad gait structures are shown as transparent curves. In all cases, skipping and asymmetrical running spanned the gap between the hopping and symmetrical running branches. The gait transitions points (triangles) spanned almost the entire speed range. Comparing **(A)**, **(B)**, and **(C)**, demonstrates that gait structure varies greatly with neutral leg swing angle offset. The shadowed region in Figure 9C is shown in Figure 11 as a comparison between simulation solutions and experimental data.

**FIGURE 10:**
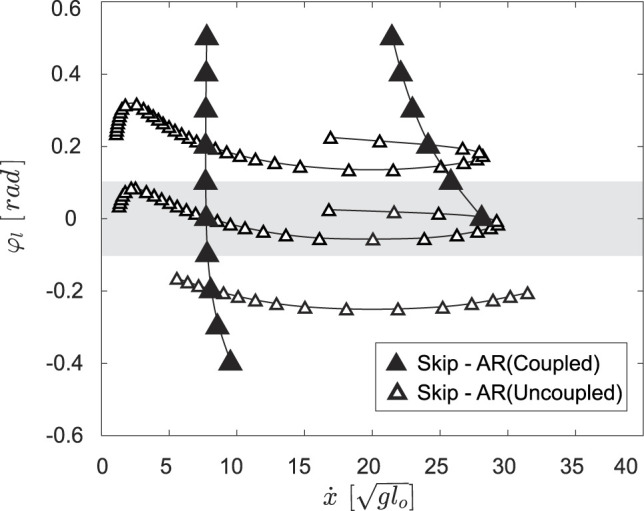
The skip - asymmetrical run (AR) transitions for the coupled model, $\varphi_{r}=\varphi_{l}\in \left[-0.5,0.5\right]$ , vary slightly with speed and occur in two narrow ranges of speed. The uncoupled model, *φ*_*r*_ =[−0.2,0,0.2], finds far more solutions for the same type of gait transition throughout a broader speed range.

Combining positive and negative variations in neutral leg angle offset shows that asymmetrical gaits spanned the gaps between symmetrical running branches (Figure 9). Thus, changing the offset between left and right neutral leg angles effectively enables transitions between symmetrical and asymmetrical gaits. Even within the asymmetrical gait structure, skipping-asymmetrical running transition points (triangles in Figure 9) spanned nearly the entire range of speed in response to small variations in neutral leg swing angles. Specifically, the forward speed of transition points varied from 1.30 to $29.26\sqrt{gl_{o}}$ , while the left leg neutral leg swing angle only varied from −0.06 to 0.14 rad (Figure 9B). In comparison to the large gaps between gait transitions in the coupled model (Section 3.3), the uncoupled model finds abundant solutions for gait transitions throughout the full range of speeds (Figure 10).

### 3.5 Validation

**FIGURE 11:**
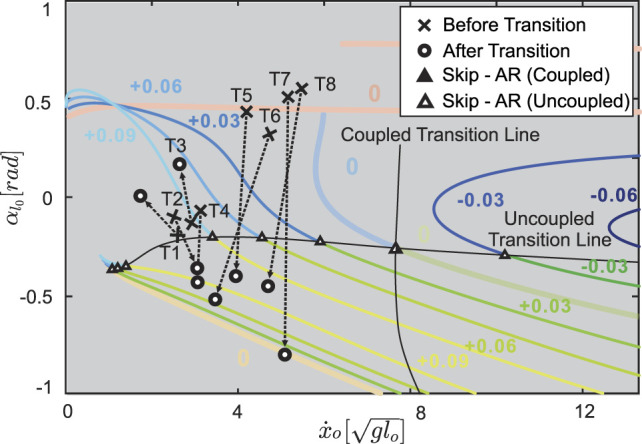
Transitions observed from the jerboa experiments (crosses to circles) in comparison to the predicted gait structure in uncoupled models (colored branches and triangles from Figure 9C) and the predicted transition lines (solid triangles represent coupled transitions and hollow triangles represent uncoupled transitions, the intersection of coupled and uncoupled transition is shown in half-solid and half-hollow). The crosses indicate the apex states before the transition, the hollowed circles are the apex state after transition, and the arrows show the transition directions on the Poincaré section. T1 to T4 show skip - asymmetrical run (AR) transitions, while T5 to T8 show transitions from hopping. The arrows pass through, or near, hollow triangles, showing that the model with the uncoupled, rather than the coupled, NLSA mechanism accurately predicts gait transitions that are observed in empirical data.

To validate our model, we compared our predictions to empirical gait transition data from jerboas. We found that jerboas swing each leg with a different, non-zero neutral leg swing angle. Specifically, jerboas tend to fix the neutral swing leg angle of one leg while varying the neutral swing leg angle of the other leg. For instance, for j38 (column 5–8 in Table 1) the neutral swing leg angle *φ*_*r*_ for its right leg was −0.08 ± 0.31 rad while *φ*_*l*_ was 0.00 ± 0.06 rad. As shown in Figure 4, with the same set of parameters, ${\vec{p}}^{T}=\left[m,l_{o},g,k,\omega ,\varphi_{l},\varphi_{r}\right]$ , our model can reproduce five bipedal gaits simply by regulating the initial states and altering the total energy. From our experimental data set, there were four gait transitions between skipping and asymmetrical running (T1 to T4 in Figure 11) and four transitions from hopping to other gaits (T5 to T8 in Figure 11). These transitions occurred when the NLSA (*φ*_*r*_) were close to 0.2 rad and were thus compared to model predictions with similar NLSA values (shaded region in Figure 9C). In both T1 and T3, neither the uncoupled nor the coupled model accurately predicts the behavior of the transition (i.e., the empirical transition line from cross to circle does not cross the model transition line). A closer examination of the video data revealed that these trials involved a jerboa decelerating to a stop, which could be a multiple - step process and is a behavior that has not been investigated by our model. The other two transitions from skipping to asymmetrical running (T2 and T4), however, clearly fell into the regions predicted by our uncoupled model and crossed the uncoupled transition line as expected. Four transitions from hopping to other gaits (T5 to T8) were observed through the range of speeds from 4.19 to 5.47 $\sqrt{gl_{o}}$ . Because of the short recording window and the large stride lengths of the jerboas at higher speeds, the apex transitions of the stride after hopping are not visible, but the kinematic data suggest that these are transitions either to skipping or asymmetrical running. All of these trials passed the gait transition line suggested by our uncoupled leg model within one stride (the black line connected through hollow triangles) rather than the coupled leg model (black line connected through the solid triangles), matching our observation that jerboas tend to uncouple leg NLSA during locomotion. These results suggest that for non - stopping behaviors, our proposed model dynamics generate biologically relevant predictions of gait transitions.

## 4 Discussion

We present the first computational model to reproduce the locomotion patterns and gait transitions of the non-cursorial jerboa. By adding a torsional spring to a unified SLIP-like model, we varied the model swing leg dynamics to match jerboa locomotion patterns. This model accurately reproduced previously described hopping, symmetrical running, and skipping gaits and enabled the formal characterization of walking and asymmetrical running gaits for the first time. The discovery of the asymmetrical running gait describes previously unused data recorded of jerboa locomotion that did not fit into the pre-existing gait categories. Furthermore, the results of this study suggest there exist two distinct mechanisms (i.e., coupled leg motions in Section 3.3 and uncoupled leg motions in Section 3.4) for gait transitions. This modeling approach can be used to shed light on the underlying dynamics of other non-cursorial or previously uncharacterized locomotion and can inform the design of robotic controllers capable of smoothly transitioning between gaits.

In the coupled leg model, the number of gait transitions and the unique pairs of gaits between which transitions can occur remain invariant to changes in neutral leg angle. Because they lie on distinct continua, symmetrical and asymmetrical gaits can only transition to gaits of the same type, rather than across types. The existing high-speed transition between asymmetrical gaits occurs at a slightly broader range of speeds when the coupled neutral leg swing angle changes. For asymmetrical gaits, all transitions involve the skipping gait; there are no smooth transitions directly between hopping and asymmetrical running.

Our model suggests that by uncoupling the motions of a leg pair, jerboas can greatly vary the range of speeds at which gait transitions can occur and introduce novel transitions between asymmetrical and symmetrical gaits. As shown in Figure 10 C, by varying the *φ*_*l*_ by merely +0.08 rad (4.6°), the speed at which the skip - asymmetrical run transition occurs increases from 0 to $7.5\;\sqrt{gl_{o}}$ . This demonstrates how at any speed, a jerboa can change its swing leg behavior and instantaneously transition to another gait pattern within one step. Moreover, changing the neutral leg angle anteriorly causing a shift of the whole gait branch to low speed regions and vice versa. This uncoupled swing leg strategy provides a mechanistic explanation for the observation that jerboas use gait transitions to quickly accelerate, decelerate, or regularize its forward speed (Moore et al., 2017). Another key observation of this study is that the skipping gait and asymmetrical running gait played critical roles in bridging the symmetrical gaits and asymmetrical gaits. For example, in Figure 8A, when the left neutral leg angle shifted anteriorly, the asymmetrical running (green curve) approached the vicinity of the running branch (opaque yellow curve). With posterior shifts in neutral leg swing angle, as shown in Figure 8B, the skipping gait (blue curves) approached the bipedal hopping gait (red transparent curve) across a broad range of speeds. Throughout this process, skipping and asymmetrical running remained on the same continuum with each other.

The results from our model reflect two mathematical definitions of gait asymmetry (Ian and Golubitsky, 1993, Chapter 8) — temporal asymmetry creates phase desynchronization between the legs (which can occur either with coupled or uncoupled changes in NLSA), while model asymmetry (e.g., uncoupled changes in NLSA) generates distinct leg behaviors. The model behaviors that arise from this mathematical distinction provide a useful framework to identify the mechanisms by which genes control motion coordination (Andersson et al., 2012).

Although previous work with conventional SLIP models succeeded in eliciting gait transitions (Geyer et al., 2006), a gait identified by providing a pre-defined leg contact angle provides no intuitive explanation for the system dynamics that generate the necessary changes in contact angle. In our proposed model, we add a torsional spring so that changes in leg contact angles become governed by the passive dynamics of the system. Thus, gait structure emerges as a result of model parameters, which provide a mechanistic explanation for the resulting gait transitions. This distinction can further enhance our understanding of animal gaits and lay the foundation for better legged robot controller design.

For example, as shown in Figure 11, in some cases (Figure 11, T4 & T7) jerboas may transition from one fixed point to another fixed point on the same gait structure. This would mean that the jerboa kept using the same set of parameters (including the same NLSAs) and only altered the total energy in a single step. In other cases (Figure 11, T3 & T5), transitions between branches would indicate that both the total energy and the NLSAs have been altered to facilitate these transitions.

Our work can also inform controller design because it suggests that we can use virtual constraints (Westervelt et al., 2018, Chapter 1) that control leg swing behavior by modeling it as a pendulum with a torsional spring. Then we can modulate the total energy in the system to accelerate, decelerate, or switch gaits, while compensating for energy losses through joint friction or collisions. One can also use our solution branches as “a lookup table” in the design of locomotion controllers as proposed in our previous work (Cnops et al., 2015). To dynamically and efficiently change locomotion pattern at any desired gait or speed, if the current states of the application are known, the controller can search for an optimal trajectory to plan either a one - step or multiple - step process without performing any expensive calculation.

Many of the solutions found in the proposed bipedal model can be directly applied to quadrupedal locomotion. According to the idea of dynamic similarity (Alexander and Jayes, 1983), when quadrupedal animals synchronize their leg motions in pairs (i.e., trotting, pacing, and bounding), the leg pair behaves as a unified leg with a greater stiffness. As discussed in our previous work (Gan et al., 2018a), the running and hopping branches in the gait structure of the bipeds are functionally identical to the trotting and pronking gaits of quadrupeds. Similarly, the shapes of skipping and asymmetrical running branches in the bipedal model will closely resemble bounding and galloping in the quadrupedal model. However, in the quadrupedal model, because legs pairs are connected to the torso at different locations, the asymmetrical gaits with different sequences of leg touchdowns will create unbalanced moments about the COM of the main body and cause the torso to rotate. As a result, the actual bounding and galloping branches of the quadrupedal model will also depend on the inertial properties of the torso. In general, when the quadrupedal model shares similar parameter values to those of the proposed bipedal model, we expect similar transitions will happen among these quadrupedal gaits, based on the gait structure shown in Section 3.2.

In our future work, we plan to extend our model by adding another pair of legs to find transitions between quadrupedal and bipedal locomotion, as observed in the escape behaviors of lizards, rodents, cockroaches, and during the locomotor development of jerboas (Marlow, 1969; Full and Tu, 1991; Eilam and Shefer, 1997; Clemente, 2014). A combined quadrupedal and bipedal model can provide novel insights into the neurological changes that likely facilitate the evolution of ephemeral and obligate bipedal locomotion.

## Data Availability

The model and results for this study can be found in the UM Deep Blue Data repository https://doi.org/10.7302/ewaa-qm16.
